# Randomized Controlled Trial of Patient Positioning and Operator Radiation Exposure During Lower Extremity Catheter Angiography

**DOI:** 10.3390/life15091433

**Published:** 2025-09-12

**Authors:** Ákos Bérczi, Fanni Éva Szablics, Anita Nelli Simon, Gabriella Taba, Dóra Ágota Papp, Réka György, Ákos András Pataki, Artúr Hüttl, Balázs Nemes, Csaba Csobay-Novák

**Affiliations:** 1 Department of Interventional Radiology, Heart and Vascular Centre, Semmelweis University, Városmajor utca 68, 1122 Budapest, Hungarycsobay.csaba@semmelweis.hu (C.C.-N.); 2 Radiation Protection Service, Semmelweis University, 1085 Budapest, Hungary

**Keywords:** chronic limb-threatening ischemia, diagnostic catheter angiography, radiation exposure, radiation safety, occupational exposure, transradial

## Abstract

Digital subtraction angiography (DSA) remains an important reference modality for evaluating chronic limb-threatening ischemia (CLTI), with left transradial access (TRA) increasingly favored for its lower complication rates and patient comfort. Radiation safety for operators is paramount, yet the impact of patient positioning on scatter radiation during lower limb diagnostic catheter angiography (CA) is understudied. This single-center randomized controlled trial evaluated whether head-first (HF) vs. feet-first (FF) supine patient orientation affects operator radiation exposure during lower extremity CA from left TRA. Between February and August 2024, 24 patients with CLTI were enrolled and randomized to HF or FF positions. Operator radiation exposure was measured using thermoluminescent dosimeters (TLDs) at the eye, chest, and left ring finger. Background radiation was subtracted. Procedures were standardized and performed by a single experienced interventional radiologist. Fluoroscopy time, dose area product (DAP), and contrast usage were recorded. No statistically significant differences were found between groups in patient BMI and procedural parameters. Patient positioning (HF vs. FF) did not significantly impact operator radiation exposure. A trend toward higher finger exposure in FF position suggests the need for optimized hand protection. These findings support flexible patient positioning without compromising operator safety, reinforcing adherence to ALARA principles.

## 1. Introduction

Imaging for chronic limb-threatening ischemia (CLTI) requires non-invasive (ultrasound, computed tomography angiography, and magnetic resonance angiography) and, in patients where revascularization is planned, minimally invasive approaches [1]. Among these, catheter angiography (CA) remains an important reference technique, as it has been proven superior in target lesion visualization for below-the-knee and below-the-ankle arteries, compared to non-invasive imaging [1,2,3]. The transradial access (TRA) for digital subtraction angiography (DSA) of the lower extremities is increasingly popular due to lower complication rates and improved patient comfort [4,5,6]. Compared to the transfemoral approach, the radial artery is more superficially located over the radial bone, allowing easier hemostasis and bleeding control. In addition, the vascular preserve provided by the ulnar artery usually prevents ischemic complications of the hand [7,8]. While right TRA is most commonly chosen in cardiac catheterization laboratories due to both operator and patient preference [9], the left-sided approach ensures direct access to the left subclavian artery, reducing procedural complexity [7]. In addition, published publications has shown lower major complication rates, reduced procedural duration, and higher rate of procedural success when using left radial access, rather than puncturing the right side [10,11].

In accordance with the ALARA (as low as reasonably achievable) principle, the radiation dose to patients, operators and staff should be minimized during X-ray exposure in angiographic suites [12]. In contrast to patients, occupational radiation exposure of the medical staff is limited by regulations to an effective dose of 20 mSv per year averaged over 5 years, with an upper limit of 50 mSv in any single year [13,14]. Within the medical field, interventional radiologists are among the most highly exposed to ionizing radiation [15,16], primarily due to scatter radiation originating from the patient’s body [17]. Despite the relatively minor proportion of the dose received by the operator and staff during a single intervention, the potential for stochastic effects resulting from cumulative exposure over many years is a significant consideration.

To date, there is a lack of data regarding how different patient positions impact the operator’s radiation exposure during lower limb diagnostic CA. Existing studies have predominantly focused on other variables, such as access routes, operator expertise, and protective measures [18,19,20,21,22]. However, the configuration of angiography suites—varying in floor space, equipment design, and X-ray tube positioning—suggests that patient positioning requiring different ergonomic adjustments from the operator, may also be a critical determinant of scatter radiation. This study addresses this gap by evaluating operator radiation exposure in different patient positions during angiography. The objective of this study is to investigate whether the positioning of patients, either head-first (HF) or feet-first (FF), in the supine position during lower extremity CA from left TRA affects the radiation dose received by the operator. The null hypothesis is that radiation exposure of the interventional radiologist will be similar in the two patient positions.

## 2. Materials and Methods

A randomized controlled trial was conducted at a tertiary vascular centre from February to August 2024. Consecutive patient inclusion was ensured to improve study reliability and reduce selection bias. The study protocol was approved by Regional and Institutional Committee of Science and Research Ethics (173/2023), and informed, written consent was obtained prior to participation. All components of this prospective study were conducted in accordance with the Declaration of Helsinki.

The sample size for the study was determined to 24 CA procedures (12 per study arm), assuming two-sided 5% statistical significance, 80% power, and a small, standardized effect size of 0.2. Although this represents a small effect size, even modest reductions in radiation exposure may be clinically relevant due to the cumulative occupational risks faced by interventional radiologists/operator. To reduce selection bias and achieve balance in the allocation of participants to positioning arms, patients were randomized using computer-generated random numbers with block randomization (block size 4). Allocation was concealed in sequentially numbered, opaque, sealed envelopes, opened immediately prior to the procedure. Blinding of the operator to patient positioning was not feasible due to the nature of the intervention. In this study, harms were defined as radiation exposure to the operator, which represents a potential occupational hazard due to its cumulative stochastic effects.

**Figure 1:**
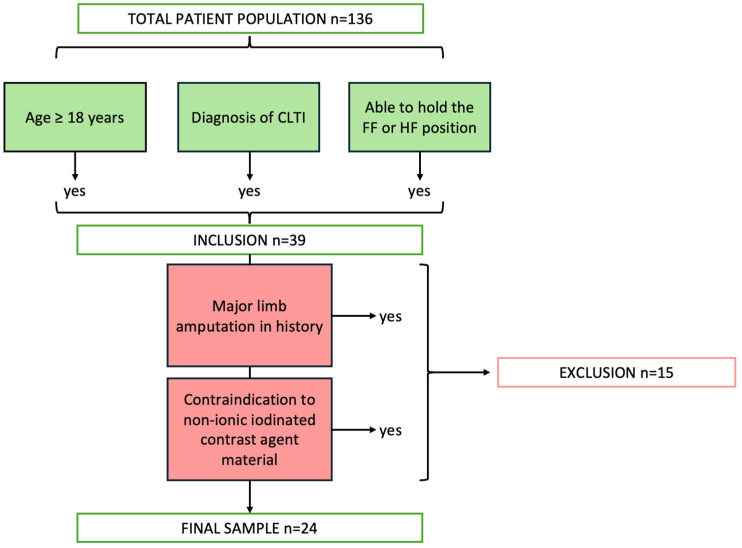
Flowchart of study participant inclusion.

To reduce treatment bias and increase homogeneity of the collected data, the following inclusion and exclusion criteria were defined (see flowchart in Figure 1):

Inclusion criteria:

- Patients with an age ≥ 18 years referred for lower extremity CA;
- Diagnosis of CLTI.

Exclusion criteria:

- Incapacity or inability to hold the FF and/or HF supine position;
- History of major limb amputation;
- Contraindication to iodinated contrast agent.

Only patients scheduled for diagnostic angiography without intervention were included.

**Figure 2:**
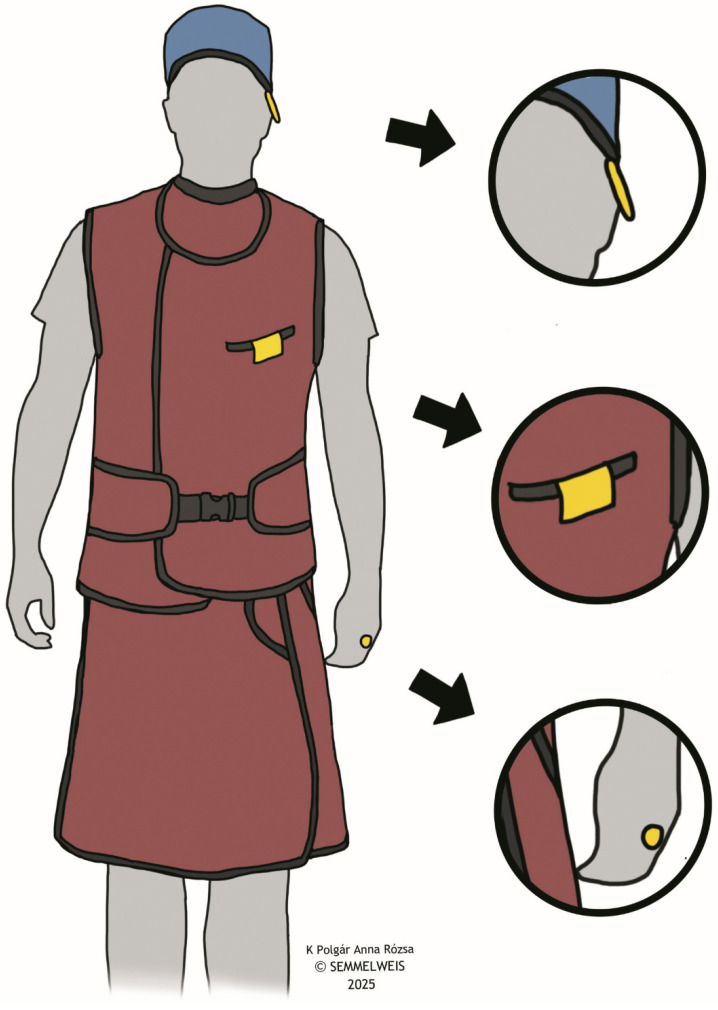
Location of the dosimeters for measuring operator doses: the left ring finger, the left temple of the lead glasses, and above the lead coat at chest level on the left side.

**Figure 3:**
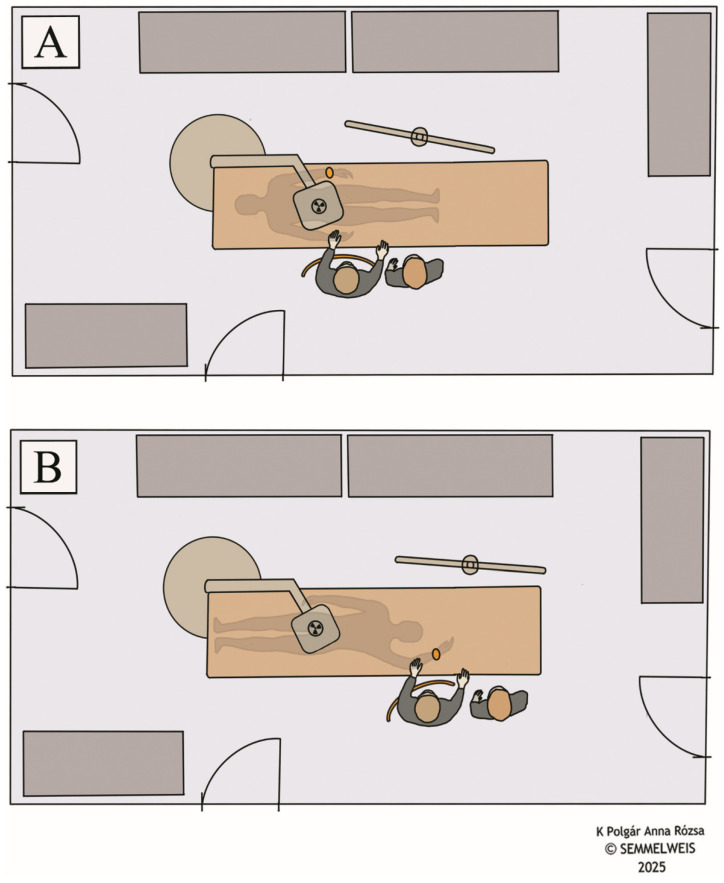
Setup of the angiographic suite, in head-first position (**A**) and feet-first position (**B**). The stand-alone moveable lead shield is visible in both figures, while the ceiling-mounted and table-mounted lead shields are not illustrated.

Given that thermoluminescent dosimeters (TLD) can have measurement errors ranging from approximately 10% to 80%, depending on various factors, there is a possibility that these errors could be comparable to or even exceed the scattered radiation doses from a single CA procedure [23,24]. Therefore, we utilized cumulative measurements to reduce the relative impact of measurement errors and improve accuracy in assessing true scattered radiation doses. Radiation doses were measured in µSv using three sets of TLDs. Specifically, two sets of TLDs were used to measure operator doses, one for each group (HF and FF). Each set included a finger dosimeter [(*H*p(0.07)], an eye dosimeter [(*H*p(3)], and a body dosimeter [(*H*p(10)], by RadPro (RadPro International GmbH, Remscheid, Germany). The dosimeters were positioned on the operator in accordance with international radiation safety protocols [25], namely on the left ring finger, the left temple of the lead glasses, and above the lead coat at chest level on the left side (Figure 2). The third set of TLDs was placed in the control room of the angiography suite for the entire study period to measure background radiation. All three sets of dosimeters were kept in the control room. When enrolling a patient, the corresponding set of TLDs was retrieved from this location, placed on the operator, and then returned to the same place immediately after use. All TLDs were calibrated prior to use by the institutional radiation safety team. Cumulative background radiation doses measured by the control room TLDs were subtracted from each operator dose reading to correct for ambient exposure. The control room radiation environment was surveyed and certified by the institutional radiation safety team to confirm compliance with shielding requirements and radiation safety standards. Evaluation of the dosimeters was performed by RadPro TLD Cube (RadPro International GmbH, Remscheid, Germany). Setup of the angiographic suite is illustrated in Figure 3, including the X-ray system and the different lead shielding. All procedures were performed using the Artis Zee with PURE angiography system (Siemens Healthineers, Erlangen, Germany), a floor-mounted monoplane C-arm system with an under-table X-ray tube and an over-table detector panel. During the procedures, stand-alone moveable lead shield and ceiling-mounted lead shield were positioned between the C-arm and the IR, as close as possible to the scatter source. A table-mounted lead shield was also in use for all procedures, besides other personal protective equipment. Specifically, a 4F sheath (Radifocus, Terumo Interventional Systems, Tokyo, Japan) was placed in the left radial artery, and a 4F Angiopointer hydrophilic angiographic catheter (APT Medical, Shenzhen, China) was advanced into the abdominal aorta for angiography. For wire exchanges, we used Accoat PTFE-coated guidewires (SP Medical, Karise, Denmark) and hydrophilic guidewires (Roadrunner UniGlide, Cook Medical, Bloomington, IN, USA). Angiography was performed on a Siemens Artis Zee with PURE (Siemens Healthineers, Erlangen, Germany) angiography system. Contrast injection was delivered with an Avanta fluid management injection system (Medrad, Warrendale, PA, USA), connected via an Angiodyn Seldinger set (B. Braun, Melsungen, Germany). Injections were made as needed to visualize the lower extremity arterial system, using automatic power injection with the operator stepping back.

In the head-first position, both upper extremities were positioned adjacent to the torso. In contrast, in the feet-first position, the left hand was elevated above the level of the head, as illustrated in Figure 3. CAs were performed from left radial access in both positions, and in accordance with the 2024 guideline of the American Heart Association and American College of Cardiology [1]. In the HF position, left radial artery access was obtained with the operator standing on the patient’s right side, reaching across the torso to puncture the left radial artery. The left wrist was propped up with slight elbow flexion and partial hand pronation, allowing stable access while maintaining patient comfort. In the FF position, the operator gained left radial artery access while standing on the patient’s left side near the head, ensuring a direct puncture approach. Patient comfort and tolerance of positioning were monitored throughout all procedures.

For vessel visualization, a non-ionic iodinated contrast agent was applied (iopromide 370 mgl/mL, ULTRAVIST^®^, Bayer AG, Leverkusen, Germany). The fluoroscopy and DSA protocols were set in accordance with the recommendations of the International Commission on Radiological Protection [13]. Fluoroscopy was taken at 7.5 frames per second. The correct would be: The DSA acquisition protocol involved a total of eight series: one posteroanterior view of the infrarenal aorta, femoral arteries, popliteal arteries, below-the-knee (BTK) arteries, and below-the-ankle (BTA) arteries; and of the pelvis, one posteroanterior view and two anterior oblique views (RAO and LAO) at a 30° angle. The DSA acquisition type was set at a pulse width of 100 ms and a dose of 3000 μGy/p, whilst the frame rate was set at two frames per second above the inguinal ligament and one frame per second for series below the inguinal ligament. The tube voltage was set to 70 kVp and was adjusted by automatic exposure control according to patient characteristics.

To create consistency across the procedures, all diagnostic procedures were performed by the same IR specialist (Á.B.), with experience of more than 100 CA/year in the last 5 years. Tube angles, table and detector height, and collimation was controlled by an interventional radiology nurse. Personal protective equipment including lead aprons, thyroid shields, and lead glasses were consistently used according to local protocols. Endovascular technique, fluoroscopy time, DSA acquisition count, and dose area product (DAP) were recorded.

Continuous variables were reported as the mean ± standard deviation (SD), compared using two-sample *t*-tests. Categorical variables were reported as percentages and compared using chi square tests or Fisher’s exact tests, as necessary. Statistical significance threshold was set at *p* < 0.05. The statistical analysis was performed on STATA software (v18.0, StataCorp LLC., College Station, TX, USA).

## 3. Results

Diagnostic CAs were performed for lower extremity arterial disease and collected dose measurements in 24 patients (mean age 71 years, ±8 SD, 14 men) altogether between February and August of 2024. All participants have been previously diagnosed with CLTI. Patients were referred to CA by a primary physician (vascular surgeon or angiologist) unrelated to the current study. Patient demography and baseline characteristics are shown in Table 1. No procedures required modification or termination, and all patients were able to tolerate both head-first (HF) and feet-first (FF) orientations. Mean duration of procedure were 5.5 (±1.83) minutes in the HF group (*n* = 12) and 6.92 (±2.43) minutes in the FF group (*n* = 12) (*p* = 0.18). Approximate mean DAP values were 1889 (±907.23) mGym^2^ in the HF group, whilst in the FF group, it was 2034.83 (±1398.48) mGym^2^ (*p* = 0.78). Mean entrance skin dose was 70 mGy in the HF group and 77 mGy in the FF group. Statistical analysis revealed no statistically significant differences between the two groups in terms of patient BMI, procedure duration, DAP, entrance skin dose, and contrast material quantities.

**Figure 4:**
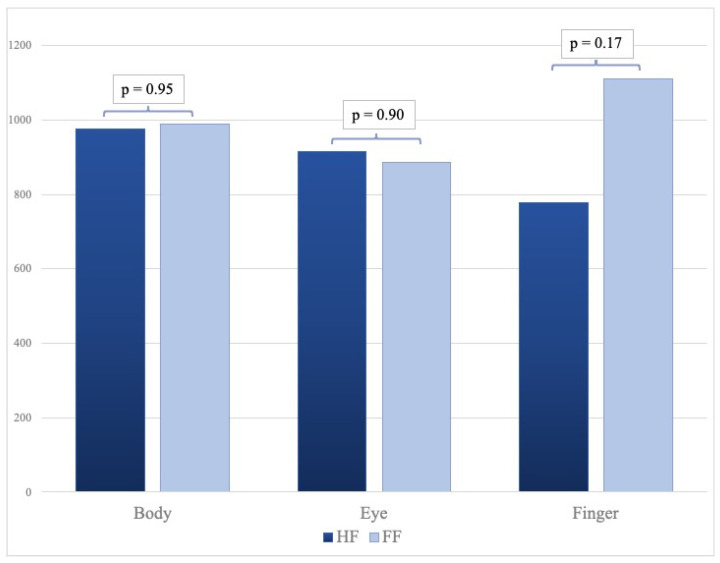
Bar graph illustrating the distribution of cumulative radiation doses (µSv) for each dosimeter in both groups, with *p*-values.

Dose for the two groups is demonstrated in Table 2. Cumulative radiation doses for the body, eye, and finger were 976 µSv, 914 µSv, and 777 µSv for the HF group, while they were 990 µSv, 887 µSv, and 1111 µSv for the FF group (Table 2). Background radiation, measured in the control room of the angiography suite was 48 µSv for finger dosimeter [(*H*p(0.07)], 93 µSv for eye dosimeter [(*H*p(3)], and 78 µSv for body dosimeter [(*H*p(10)]. To assess the relative dispersion of radiation dose measurements, we calculated the coefficient of variation (CV) for each dosimeter value, using data from previous publications. The CV is defined as the ratio of the standard deviation to the mean (CV = *σ*/*μ*), providing a normalized measure of variability independent of absolute values. No statistically significant differences were observed between the two groups (body radiation dose: *p* = 0.95, eye radiation dose: *p* = 0.90, finger radiation dose: *p* = 0.17). As demonstrated in Figure 4, the bar graph offers an illustration of the distribution of cumulative radiation doses for each TLD in both groups, including the CV for all dosimeters.

## 4. Discussion

This study demonstrates that the choice of patient positioning, whether head-first or feet-first, during lower extremity diagnostic CA does not lead to a significant difference in the operator’s radiation exposure. The measurements taken at the body, eye, and finger sites revealed comparable doses between the two groups, with no statistically significant differences. However, a notable observation of increased finger dosimetry in the FF position may reflect procedural or anatomical factors influencing operator proximity to the radiation source.

The findings align with prior studies, which have emphasized that factors such as equipment settings, operator technique, and protective measures are more critical determinants of radiation exposure than patient positioning. In our study, the similarity of the DAP and entrance dose values, supported by individual measurements and statistical analysis, suggests that the interventions were comparable. This finding confirms that randomization was effective in ensuring homogeneity between groups. Any observed differences are likely attributable to differences in staff positioning and personal radiation protection measures rather than procedural discrepancies. Forbrig et al. demonstrated that the radiation dose and fluoroscopy time are influenced to a greater degree by procedural complexity and technical considerations than by patient position during modern endovascular treatments [21]. Leyton et al. established a strong correlation between patient entrance dose and scatter radiation at the operator’s eye height, underscoring the influence of C-arm angulations and procedural complexity on radiation distribution [18]. Monastiriotis et al. reported similar findings in endovascular aneurysm repair, highlighting the importance of shielding and operator distance from scatter radiation [20]. In a quantitative observational study by Kuriyama et al., investigating the factors affecting occupational radiation exposure to the eye among nurses also found that distance from the X-ray field was the strongest determinant in eye doses [26]. Another study investigated the impact of different X-ray tube angulations on occupational radiation in cardiac angiography, and found that radiation exposure was strongly dependent on projection angle [27]. Similarly, a study published in the European Journal of Cardiovascular Nursing, in 2022, compared radial and femoral access in coronary interventions, emphasized access-related variables over patient orientation [22]. Though statistically not significant, the increased finger exposure in the FF group may correspond to observations in scatter radiation studies, such as that by Yang et al., where procedural angles and arm positioning influenced dose distribution [19].

Radiation protection measures, including optimized shielding and use of radioprotective equipment, remain of critical importance in interventional radiology. While operators and the angiographic staff need to protect their chest, hands, and head and neck region with the use of lead apron and protective eyewear (i.e., lead glasses), optimizing the C-arm angulations, distance from the radiation source, and duration of procedures are key for effective radiation protection [28,29]. Alternative approaches for minimizing operator radiation exposure, such as the use of automatic power injectors with the operator stepping back or leaving the angiography suite, have been shown to substantially reduce occupational exposure [30]. However, this technique was associated with longer procedural times and higher patient radiation doses (DAP) in lower extremity angiographies, which may paradoxically increase patient exposure. In addition, power injection carries a risk of vessel injury in distal vessels such as below-the-knee and below-the-ankle arteries, making manual injection preferable. While in our clinical practice we also step out during injections whenever feasible, this is not always possible in complex CLTI cases, where challenging anatomy requires the operator to remain in the room. A considerable part of scatter radiation arises from fluoroscopy rather than from DSA injections, and eliminating this component would only be feasible with robotic-assisted systems, which were not available in our setting. Furthermore, in our study, scatter contributions from DSA and fluoroscopy could not be separated, and their combined levels were frequently below the detection limit of individual TLDs, which necessitated pooling of patient data.

Another practical aspect to consider is patient comfort. In our study, only patients capable of maintaining both orientations were included. While all patients tolerated both without relevant discomfort, feet-first positioning may pose challenges, particularly in individuals with restricted shoulder mobility or musculoskeletal limitations. Such factors should be taken into account when selecting patient orientation, as intolerance could limit applicability in routine clinical practice.

This study has limitations, including its small sample size and single-center design, which may limit generalizability. Procedural standardization, while ensuring consistency, may not fully represent the variability encountered in routine practice. Specifically, measurements were limited to a single operator and specific angiographic equipment configuration to standardize technique. However, this may limit the external validity of our findings, as radiation doses could vary with different operators, experience levels, and across different angiographic setups. Furthermore, purely diagnostic angiograms are less common in routine clinical practice, and thus, our findings may not fully translate to longer interventional procedures. Nonetheless, this controlled design allowed isolation of the variable of interest, namely patient positioning. Differences in operator ergonomics, such as distance and angle relative to the irradiation field, and hand positioning, may contribute to altered scatter radiation between patient positions. However, this study focused on evaluating the overall impact of patient positioning itself and did not directly measure or record quantitative ergonomic parameters. As a result, while our findings reflect the cumulative effect of patient positioning in a standardized setup, they do not allow conclusions about specific contribution of each variable, underscoring the need for future research involving detailed ergonomic measurements to characterize the impact of these effects. Moreover, the directional sensitivity of the TLDs may have introduced additional measurement variability, as minor differences in the angle due to variations in operator posture and hand orientation between HF and FF patient positions could have influenced the recorded dose values. For this reason, we utilized cumulative measurements, thereby reducing the impact of such factors on scattered radiation values. Regarding the background radiation measurements in the control room, the recorded values were higher as typically expected, which is an important consideration when interpreting our results. This most likely reflects the cumulative ambient exposure over the extended measurement period. While background correction was applied to mitigate TLD measurement errors, this approach introduces an inherent limitation, as it may not perfectly reflect real-time procedural background radiation levels. Therefore, future studies should consider shorter measurement intervals or real-time dosimetry. Overall, these limitations were mitigated by using validated dosimetry methods and standardized procedural protocols. Future studies should include larger, multi-center cohorts, multiple operators, and different angiographic setups, and assess longer, interventional procedures to validate these findings and explore their broader applicability.

In conclusion, patient positioning in HF or FF supine orientation does not significantly affect operator radiation exposure during diagnostic CA. These results highlight the flexibility in patient positioning without compromising operator safety, allowing procedural ergonomics and patient-specific factors to guide positioning decisions. The observed trend of increased finger exposure in the FF group underscores the importance of optimizing shielding and hand positioning to further minimize radiation risk. This study supports the ongoing refinement of radiation safety practices in interventional radiology, ensuring compliance with the ALARA principle while maintaining clinical efficacy.

## Figures and Tables

**Table 1:** Patient demographics and procedural characteristics.

Variable	HF Mean (±SD)	FF Mean (±SD)	*p*-Value
Height (cm)	168 (6.4)	168 (9.6)	0.98
Weight (kg)	84.2 (21.14)	79.42 (23.37)	0.67
BMI (kg/m)	29.73 (6.56)	27.74 (5.69)	0.44
Duration of procedure (min)	5.50 (1.83)	6.92 (2.43)	0.18
Fluoroscopy time (s)	118.3 (34.56)	160.75 (100.43)	0.23
Dose area product (mGym^2^)	1889.00 (907.23)	2034.83 (1398.48)	0.80
Entrance skin dose (mGy)	69.95 (40.34)	77.13 (61.07)	0.77
Contrast volume (ml)	112.33 (24.87)	117 (16.71)	0.65

**Table 2:** Radiation exposure data for FF and HF groups, and standard deviation using the coefficient of variation.

TLD Position	Head-First (±SD)	Feet-First (±SD)	*p*-Value
Body (µSv)	976 (586)	990 (594)	0.95
Eye (µSv)	914 (548)	887 (532)	0.90
Finger (µSv)	777 (466)	1111 (667)	0.17

## Data Availability

The original contributions presented in this study are included in the article. Further inquiries can be directed to the corresponding author.

## Abbreviations

The following abbreviations are used in this manuscript:

DSA	Digital subtraction angiography
CLTI	Chronic limb-threatening ischemia
TRA	Transradial access
CA	Catheter angiography
HF	Head-first
FF	Feet-first
TLD	Thermoluminescent dosimeters
DAP	Dose area product
ALARA	As low as reasonably achievable
